# A hint from zebrafish models of hepatic tumorigenesis: Targeting stearoyl-CoA desaturase

**DOI:** 10.18632/oncotarget.26645

**Published:** 2019-01-29

**Authors:** Kunpeng Lv, Fei Fei, Xu Wang

**Affiliations:** Xu Wang: Key Laboratory of Metabolism and Molecular Medicine, Ministry of Education, Department of Biochemistry and Molecular Biology, School of Basic Medical Sciences, Fudan University, Shanghai, China

**Keywords:** hepatic tumorigenesis, Wnt, Ras, Scd, zebrafish

Hepatocellular carcinoma (HCC) is the second leading cause of cancer-related death and is one of major regional diseases in China [1]. Given the decades of advancement in surgery, targeted drug, and immunotherapy, the overall survival and prognosis of HCC patients remains disappointing [2, 3]. Therefore, there is an urgent need to find novel therapeutic targets of HCC.

In comparison with normal tissues, cancerous tissues may switch and modify substrate preferences for energy metabolism at both genetic level and cellular level (microenvironment) [4]. The liver is the center organ of energy metabolism, especially for lipid. A recent study reported a negative correlation of Wnt/Myc activity with ectopic steatosis in human HCC biopsy and systematically analyzed the underlying mechanisms study using three conditional transgenic zebrafish models (Wnt, Kras, Myc) [5]. Those tiny zebrafish models employed a hepatocyte-specific promoter *fabp10a*, doxycycline-inducible TetOn system and/or chimeric Gal4-UAS system to drive the tissue-specific expression of human mutant *CTNNB1*, mouse *Myc*, zebrafish *tcf7l2* in a dosage-dependent manner. The small size and transparency allowed researchers to delicately monitor the morphological dynamics *in vivo* in real time, and different combinations of oncogenic insults produced distinct phenotype in term of lipid accumulation. In addition, since the yolk serves as the only nutrient source of the zebrafish models at early larval stages, the tumorigenesis process and molecular indexes are highly-constant among individuals. Lipidomics and transcriptomics were then introduced to dissect the transformation of lipid metabolism, and one significant observation was that hepatic Ras activity induces the accumulation of large amounts of triglycerides (TGs), while additional Wnt or Myc activity converts Tgs into diacylglycerols (DGs) and phospholipids that are crucial components of membrane structures. Interestingly, the length and saturation of the side chain fatty acids of certain lipids vary, and inhibition of stearoyl-CoA desaturase (SCD), either in genetics or using small molecular drugs, significantly repressed the tumorigenesis driven by different oncogenic events.

In consistent with the findings in zebrafish models of hepatic tumorigenesis, studies from mammalian systems also strengthen the potential value of using SCD as a novel target of HCC. In hepatic tumorigenesis, the Wnt signaling pathway increases sterol regulatory element binding protein 1 (SREBP1)-dependent transcription of *Scd*, and multi-unsaturated fatty acids (MUFAs) produced by SCD stabilize β-catenin and mRNAs encoding low density lipoprotein receptor-related proteins 5 and 6 (Lrp5/6) as a positive feedback loop, promoting the tumorigenesis [6]. SCD was also found to play key role in regulating liver cancer stem cell, self-renewal, metastasis and sorafenib resistance *via* the ER stress-induced unfolded protein response. The combination of SCD inhibitor and sorafenib achieved improved efficiency in treating xenograft mice models [7].

However, SCD is the key enzyme catalyzing the synthesis of monounsaturated fatty acids (MUFAs), oleate (C18:1) and palmitoleate (C16:1). Long-term inhibition of SCD may damage the lipid metabolism in normal tissues, result in malnutrition and harm the therapeutic outcome of anti-cancer treatment. Zebrafish larvae are good systems for high-throughput screening system [8]. To compromise these side effects, a free fatty acids replenishment screening on the zebrafish models of HCC at *scd* homozygous mutant backgrounds may be performed, with a hope to identify a precision combination of the supplementary fatty acids that are beneficial to normal tissues but useless for cancerous tissues.
